# Direct purification of detergent-insoluble membranes from *Medicago truncatula* root microsomes: comparison between floatation and sedimentation

**DOI:** 10.1186/s12870-014-0255-x

**Published:** 2014-09-30

**Authors:** Christelle Guillier, Jean-Luc Cacas, Ghislaine Recorbet, Nicolas Deprêtre, Arnaud Mounier, Sébastien Mongrand, Françoise Simon-Plas, Daniel Wipf, Eliane Dumas-Gaudot

**Affiliations:** UMR1347 INRA/Agrosup/Université de Bourgogne Agroécologie, Pôle Interactions Plantes-Microorganismes - ERL 6300 CNRS, 17 Rue Sully, BP 86510, F-21065 Dijon Cedex, France; CNRS, Laboratoire de Biogenèse Membranaire (LBM), Université Bordeaux UMR 5200, F-33000 Villenave d’Ornon, France; UMR CSGA: Centre des Sciences du Goût et de l’alimentation, UMR 6265 CNRS, 1324 INRA-uB, Dijon, France

**Keywords:** Detergent insoluble membrane, Proteomic, Plant microdomain, Microsomes, Organelles, Medicago truncatula

## Abstract

**Background:**

Membrane microdomains are defined as highly dynamic, sterol- and sphingolipid-enriched domains that resist to solubilization by non-ionic detergents. In plants, these so-called Detergent Insoluble Membrane (DIM) fractions have been isolated from plasma membrane by using conventional ultracentrifugation on density gradient (G). In animals, a rapid (R) protocol, based on sedimentation at low speed, which avoids the time-consuming sucrose gradient, has also been developed to recover DIMs from microsomes as starting material. In the current study, we sought to compare the ability of the Rapid protocol *versus* the Gradient one for isolating DIMs directly from microsomes of *M. truncatula* roots. For that purpose, Triton X-100 detergent-insoluble fractions recovered with the two methods were analyzed and compared for their sterol/sphingolipid content and proteome profiles.

**Results:**

Inferred from sterol enrichment, presence of typical sphingolipid long-chain bases from plants and canonical DIM protein markers, the possibility to prepare DIMs from *M. truncatula* root microsomes was confirmed both for the Rapid and Gradient protocols. Contrary to sphingolipids, the sterol and protein profiles of DIMs were found to depend on the method used. Namely, DIM fractions were differentially enriched in spinasterol and only shared 39% of common proteins as assessed by GeLC-MS/MS profiling. Quantitative analysis of protein indicated that each purification procedure generated a specific subset of DIM-enriched proteins from *Medicago* root microsomes. Remarkably, these two proteomes were found to display specific cellular localizations and biological functions. *In silico* analysis of membrane-associative features within R- and G-enriched proteins, relative to microsomes, showed that the most noticeable difference between the two proteomes corresponded to an increase in the proportion of predicted signal peptide-containing proteins after sedimentation (R) compared to its decrease after floatation (G), suggesting that secreted proteins likely contribute to the specificity of the R-DIM proteome.

**Conclusions:**

Even though microsomes were used as initial material, we showed that the protein composition of the G-DIM fraction still mostly mirrored that of plasmalemma-originating DIMs conventionally retrieved by floatation. In parallel, the possibility to isolate by low speed sedimentation DIM fractions that seem to target the late secretory pathway supports the existence of plant microdomains in other organelles.

**Electronic supplementary material:**

The online version of this article (doi:10.1186/s12870-014-0255-x) contains supplementary material, which is available to authorized users.

## Background

Biological membranes that compartmentalize cells into organelles or form a barrier to the outside environment are composed of lipids as well as a variety of *trans*-membrane, lipid-modified and lipid-associated proteins essentially involved in transport, signaling, differentiation and stress adaptation processes. Aside from the fluid mosaic model that refers to a homogenous distribution of lipids and proteins within the plasma membrane (PM) [1], a large body of evidence supports the microdomain hypothesis [2], stating that membranes are also compartmentalized by uneven distributions of specific lipids and proteins into microdomains termed membrane rafts. Originally characterized in animal and yeast cells, membrane rafts are defined as plasma membrane [1] nano- or microdomains enriched in sphingolipids and sterols, which act as platforms initiating signaling events in diverse physiological situations, including inflammation processes and apoptotic cell death [3]. The main hypothesis relative to the functional significance of these domains relies on the lateral segregation of membrane proteins that creates a dynamic scaffold to organize particular cellular processes [4]. In plants, sphingolipid- and sterol-enriched membrane microdomains were also isolated from PM. Characterization of their protein content revealed their enrichment in proteins involved in signaling and response to biotic/abiotic stresses [5-8], suggesting that plant membrane microdomains may exert similar signaling functions to their animal counterparts.

Due to their enrichment in sphingolipids and sterols, membrane rafts form tight packing liquid-ordered (Lo) phases that segregate from the rest of the PM. An increased resistance to solubilization by detergents of Lo *versus* liquid-disordered (Ld) phases has led researchers to consider that membrane fractions insoluble to non-ionic detergents at low temperatures could contain the putative raft fractions. One caveat of this theory is that recovered detergent-insoluble membrane (DIM) fractions only exist after detergent treatment, and do not correspond to the native membrane structure [9]. Nevertheless, their significant enrichment in sterols, sphingolipids and specific subsets of proteins, some of which displaying a clustered distribution within the PM [10], has encouraged their use as a biochemical counterpart of Lo microdomains existing in biological membranes. From an experimental perspective, upon detergent application to PM-enriched preparations, DIM fractions are usually purified by ultracentrifugation onto a sucrose gradient and appear as a ring floating at low density, which are structurally represented by vesicles and membranes sheets [5]. Initially, microdomains were thought to be exclusively present in PM and membranes belonging to the late secretory pathway [11]. As indicated in Table 1*,* most of DIM preparations were indeed carried out using PM-enriched fractions as starting material [5-7,12-15], thus hampering their identification within other cell membranes. The presence of raft-like regions within organelles was nonetheless further suggested to occur upon the characterization of DIMs extracted from membranes of Golgi complex [16], mitochondrion [17] and vacuole [18,19]. To date, the widest investigation addressing the intracellular distribution of plant DIMs has been performed in Arabidopsis using whole cell membranes originating from liquid root callus cultures [20]. Noteworthy, the results obtained strongly suggested that in *A. thaliana* roots, DIMs are predominantly derived from PM sphingolipid- and sterol-rich microdomains by virtue of their substantial depletion of intracellular organelle proteins.

**Table 1:** **Main literature background to microdomain preparations as related to initial fractions**

**Organism**	**Organ/Culture**	**1st fraction**	**2nd fraction**	**Detergent**	**DIM recovery process**	**References**
Tobacco	Leaves	Microsomes	PM	Triton X-100	F	[12]
Tobacco	Leaves	Microsomes	PM	Triton X-100	F	[5]
**Arabidopsis**	**Root callus cultures**	**Microsomes**	**None**	**Triton X-100**	**F**	**[** 20 **]**
Tobacco	Cell cultures	Microsomes	PM	Triton X-100	F	[6]
Tobacco	Cell cultures	Microsomes	PM	Triton X-100	F	[7]
Leek and Arabidopsis	Seedlings	Microsomes	GA, ER, PM	Triton X-100	F	[16]
Medicago	Roots	Microsomes	PM	Triton X-100	F	[13]
**Human**	**Cell cultures**	**Cell pellets**	**None**	**Triton X-100**	**S**	**[** 25 **]**
Human	Cell cultures	Mitochondria	Mmito	Triton X-114	S	[17]
Arabidopsis	Cell cultures	Microsomes	PM	Triton X-100	F	[14]
Oat and Rye	Leaves	Microsomes	PM	Triton X-100	F	[15]
Red beet	Roots	Vacuoles	Tonoplast	Triton X-100	F	[18]
Arabidopsis	Cell cultures	Vacuoles	Tonoplast	Triton X-100	F	[19]

Organelle *versus* microsomes and DIM recovery processes: floatation on sucrose gradient (F) *versus* sedimentation (S). GA, ER, Mmito and PM, and refer to Golgi apparatus, endoplamic reticulum, mitochondrial membrane and plasma membrane, respectively. Bold characters highlight the two protocols used in the current study.

Whether this result also holds true for plants of agronomic has not been investigated yet, despite the recognized importance of membrane microdomains during plant-microbe interactions (reviewed in [4,8]). Although *Medicago truncatula* has been retained more than ten years ago as the model for studying legumes and root symbiotic interactions with fungi and bacteria [21], only one report has been dedicated to the analysis of DIM fractions in barrel medic [13]. The study showed that membrane raft domains corresponding to Triton X-100 insoluble membranes could be obtained from *M. truncatula* root PM. Additionally, evidence was given for their enrichment in proteins associated with signaling, cellular trafficking and redox processes. A raft protein termed Symbiotic REM (MtSYMREM1, or MtREM2.2) [22] was also found to control *Sinorhizobium meliloti* infection as well as rhizobial release into host cell cytoplasm within root symbiotic structures, the so-called nodules [23]. Likewise, Haney and Long [24] identified two microdomain-associated plant flotillins required for infection by nitrogen-fixing bacteria. These data raise the possibility that rafts may be involved in molecular events leading to successful nodule onset, and it is tempting to speculate that additional symbiotic associations like mycorrhiza may also require proper raft structures for their establishment and functioning. Elucidating microdomain function(s) in symbiosis and legume physiology thereby implies increasing knowledge about their cellular distribution coupled to fast and efficient methods dedicated to their isolation.

Although DIM fractions have been successfully prepared from *M. truncatula* root tissues using PM as starting material [13], this protocol requires a huge amount of root tissues. Additionally, purifying PM fractions turns out to be somehow labor-intensive and time-consuming. To overcome these technical limitations together with enlarging the coverage of DIM populations in legume roots, we investigated in the current study an alternative that relies on the possibility to skip the PM fractionation step, to isolate DIM fractions directly from microsomes, as previously described in other animal and plant model systems (Table 1). This work was thus intended to purify microdomains directly from *M. truncatula* root whole cell membranes by comparing two fast protocols previously described for DIM purification [20,25]. Using roots of soil-grown *M. truncatula* plants as starting material, we first analyzed the impact of detergent final concentration and detergent/protein ratio on lipid and protein patterns of DIM fractions. We then selected specific experimental conditions and used a GeLC-MS/MS proteomic approach, where biological samples are separated by SDS-PAGE, sliced, digested in-gel and analyzed by LC-MS/MS, on the DIM fractions retrieved from the two distinct protocols. Respective DIM protein populations were further contrasted with regard to their functional and cellular distributions.

## Results and discussion

### Purification of DIMs from *M. truncatula* root microsomes

**Figure 1:**
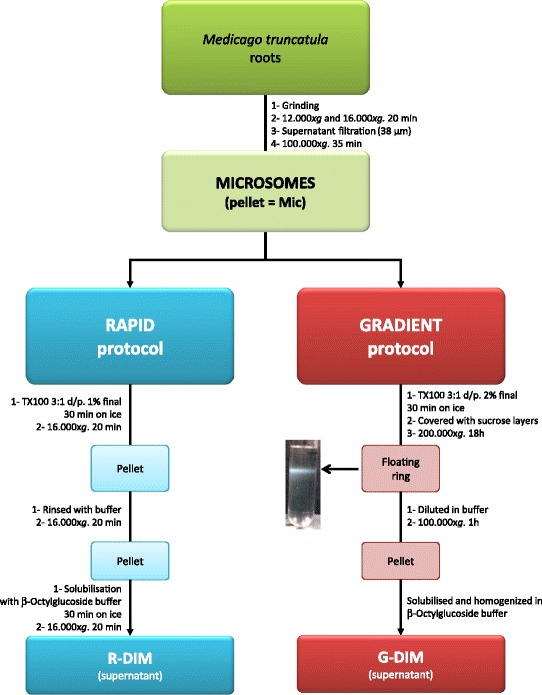
**Overview of the Rapid and Gradient protocols used for isolating DIM fractions from** ***M. truncatula*** **roots microsomes.**

In the current study, whole root cell membranes from soil-grown plants were first extracted according to the differential centrifugation-based strategy initially developed for *Nicotiana tabacum* cell cultures [7]. DIMs were further isolated from the root microsomal fraction according to two distinct protocols. The former developed by Adam and collaborators [25] consists of a rapid method for purifying DIM fractions from human cells by low speed sedimentation that exploits the differential solubility of detergent-resistant microdomains in cold, non-ionic detergents. Briefly, upon cell mechanical disruption, the authors directly treated homogenates with cold Triton X-100 (TX-100) and centrifuged samples to recover detergent-insoluble material in the pellet. These DIMs were then solubilized using β-octylglucoside as detergent and the resulting supernatant recovered after centrifugation. This procedure referred to as Rapid or R-protocol, was compared to that used by Borner *et al.* [20], which is classical floatation of cell extracts in a sucrose density Gradient (G), as illustrated in Figure 1. The latter, initially carried out using Arabidopsis callus cultures, relies on the light buoyant density of TX-100-insoluble microsomal membranes. R- and G-DIM subsets were thus prepared as explained in the section “Methods” and subsequently analyzed for their lipid and protein composition relative to the original microsomal fraction. Additionally, considering that R- and G-DIM extraction methods relied on the use of distinct TX-100/protein ratios and TX-100 final concentrations (R3:1 and G3:2, respectively), the effects of detergent-to-protein ratio (w/w) and detergent final concentration (% v/v) on lipid and protein composition were also investigated.

### Sterols, but not sphingolipids, are differentially enriched between R- and G-DIM fractions

**Figure 2:**
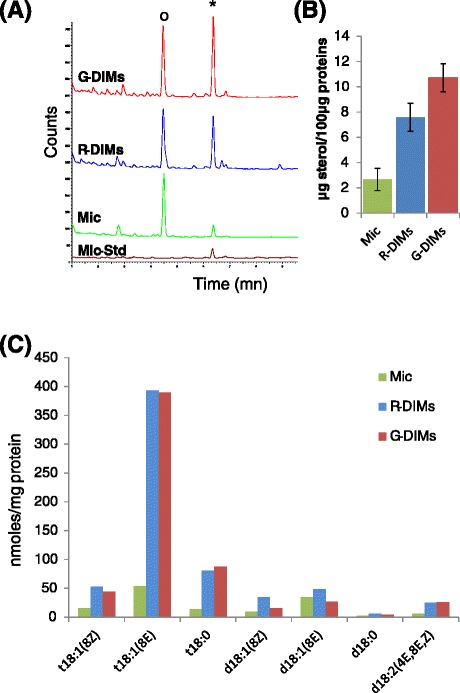
**Comparison of sterol and long-chain base (LCB) contents in R-DIMs(R) and G-DIMs (G) relative to the initial microsomal (Mic) fraction of** ***M. truncatula*** **roots.** All experiments were performed with 100 μg protein equivalents **(A)** Representative GC profile of total extracted sterols in the three fractions. Epichoprostanol (O) was used as an internal standard (10 μg) to quantify major sterol peaks (*) and added to the Mic, R and G fractions but not to the Mic minus Std (Mic-Std) sample. **(B)** Sterol enrichment from 100 μg protein equivalents. Results are expressed as the means ± SE (vertical bars) of at least three independent preparations. **(C)** Representative distribution of LCB in the three fractions; Abbreviations used: t18:1(8Z): 4-hydroxysphing-8(Z)-enine, t18:1(8E): 4-hydroxysphing-8(E)-enine, t18:0: 4-hydroxysphinganine (phytosphingosine), d18:1(8Z): sphing-8(Z)-enine, d18:1(8E): sphing-8(E)-enine, d18:0: sphinganine (dihydrosphingosine), d18:2(4E,8E,Z): sphinga-4(E),8(E,Z)-dienine.

Three independent experiments were performed and both R- and G-DIM fractions were examined for their lipid content in order to assess enrichment in sterols and sphingolipids, a typical feature of membrane microdomains. Sterol composition was first determined by gas chromatography (GC) using epichoprostanol as internal standard. Figure 2A shows a representative elution profile for R- and G-DIM fractions, relative to the microsomal (Mic) set used as starting material for DIM purification. In accordance with previous data [13], spinasterol was recorded as the most abundant sterol in the two DIM fractions, but average enrichment-fold in spinasterol increased from 2.8 to 3.9 in R- and G-DIMs, respectively (Figure 2B). Due to the distinct TX-100/protein ratios and TX-100 final concentrations published for R- and G-DIM extractions, spinasterol content was thus quantified in relation to these two parameters. At identical TX-100/protein ratios and final TX-100 concentrations, significant differences in spinasterol enrichment were still registered between R- and G-DIM fractions (Additional file 1: Figure A1A). These results clearly indicated that respective purification steps of R- and G-methods, i.e. low speed centrifugation *versus* sucrose gradient, were responsible for differences in spinasterol concentration, irrespective of TX-100-related parameters.

As long-chain base (LCB) represent a common backbone to all sphingolipids, they were quantified by GC-MS [26] as a way to access the total enrichment in sphingolipids in R- and G-DIM fractions. Whatever the method used for DIM preparation, the resulting total LCB composition (Figure 2C) was consistent with previously data reported for *M. truncatula* [13], even though there was evidence for additional minor dihydroxylated LCB (d18:0, d18:1 and d18:2), the detection of which was previously ascribed to the high sensitivity of GC-MS [26]. Interestingly, both R- and G-fractions were highly enriched in trihydroxylated LCB (c.a. 6-fold increase in t18:0 and t18:1 when compared to Mic). These compounds are mainly found amidified in the sphingolipid class of glycosyl-inositolphosphoryl-ceramides [27]. Additionally, R- and G-samples also exhibited a very similar LCB profile with identical enrichment-folds whatever the TX-100 concentration used (Additional file 1: Additional A1B), strongly suggesting that sphingolipid content is not dependent on the method used for DIM isolation. Overall, the lipid composition of R- and G-DIMs confirmed their enrichment in sphingolipids and sterols relative to the microsomal fraction.

### DIM protein composition is impacted by the extraction method

**Figure 3:**
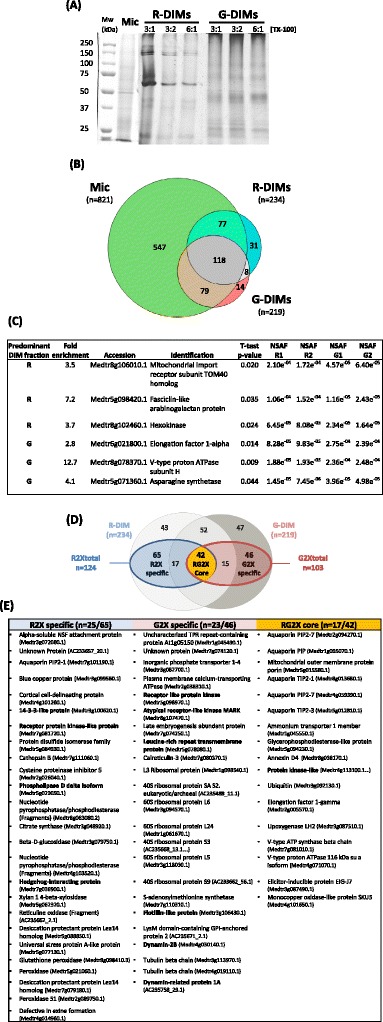
**Comparison of the proteins identified in R- and G-DIMs relative the initial microsomal (Mic) fraction of** ***M. truncatula*** **roots. (A)** One dimensional profile of the proteins (15 μg per lane) recovered in R (R) and G-DIMs (G) using variable Triton X-100 concentrations: 3:1, 3:2, 6:1 and 6:2 (detergent/protein ratio: final detergent concentration). **(B)** Venn diagram distribution of the 874 non redundant proteins overall identified using GeLC-MS/MS in the microsomal, R- and G-DIM fractions. **(C)** List of the proteins that display a differential accumulation (p < 0.05) between R- and G-DIMs. Comparison of protein abundance was performed using the Student’s *t*-test on arsin square root-transformed normalized spectral abundance factors (NSAF). NSAF ratios of proteins between the two DIM fractions are provided in column 2. **(D)** Venn diagram distribution of the 227 proteins that reproducibly display at least a 2-fold higher abundance in DIM fractions than in microsomes. Subsets termed “R2xspecific” and “G2xspecific” refer to the proteins uniquely enriched in R-and G-DIMs, respectively, whereas “RG2xcore” designates the proteins enriched in both R- and G-DIMs, relative to microsomes. **(E)** Representation of previously published plant DIM-associated proteins within the proteins enriched in R- and G-DIMs relative to microsomes, by using identification mapping tools and homology search. Bold characters refer to canonical plant DIM markers.

Due to differences in the original setups for TX-100 concentrations between R and G protocols, the effects of detergent concentration and detergent/protein ratio (ratio detergent/protein = 3 to 6 and final concentrations 1 to 2) on DIM protein composition were also preliminary assessed on the basis of one-dimensional SDS-PAGE banding patterns visualized following Coomassie blue staining. As displayed in Figure 3A, the protein profiles obtained for R-DIM samples looked different from the microsomal fraction from which they originated, but roughly qualitatively similar in the conditions of interest (R3:1 and R3:2). Despite some minor differences, increasing the ratio d:p to 6:1 did not change drastically the protein pattern. These observations also hold true for G-DIM samples. By contrast, there were noticeable qualitative and quantitative differences in protein patterns between R- and G-fractions, indicating that in our experimental conditions DIM protein contents largely depends on the isolation process rather than detergent concentration.

To go further in analyzing and comparing the proteins co-extracted with sterol-enriched DIM fractions, a shotgun proteomic approach was performed using the original setup for TX-100 concentrations, namely R3:1 and G3:2 conditions after admitting the detergent-independence of DIM lipid and protein composition over this range. Due to the limitations of two-dimensional electrophoresis to resolve integral membrane proteins [28], 1D gel coupled to liquid chromatography-tandem mass spectrometry (GeLC-MS/MS) was chosen to investigate the protein composition of DIM fractions. This workflow that combines a size-based protein separation to an in-gel digestion of the resulting fractions proved to be successful in expanding the coverage of membrane proteins in *M. truncatula* roots [29,30], and is also amenable to relative protein quantification methods such as spectral counting [31].

GeLC-MS/MS was thus conducted on two independently-extracted sets of R- and G-DIMs and the initial root microsomal fraction. Using a probability of peptide misidentification inferior to 0.05, a total of 874 non redundant proteins were overall identified in the microsomal and DIM fractions when retaining only those co-identified in the two replicates of each DIM, as listed in additional data (Additional file 2: Table A1). The Venn diagram distribution of microsomal, R- and G-DIM proteins, displayed in Figure 3B, indicated that relative to the 821 accessions initially identified in the microsomal fraction, R- and G-DIMs encompassed a rather similar number of proteins corresponding to 234 and 219 accessions, respectively. Although most of DIM-associated proteins (84%) were as expected also present in the original microsomal fraction, 53 accessions (16%) were uniquely identified in DIMs, indicating that the experimental procedure has enabled the identification of minor proteins that have escaped detection during mass analysis of whole membranes but are revealed upon fractionation. Noticeably, comparison of R- and G-DIMs showed that a common pool of 126 proteins was shared between both fractions, thereby defining a conserved core-set of DIM-associated proteins that overall represented 15% of the root microsomal proteome of *M. truncatula*.

To investigate whether there might be a difference in the quantitative distribution of these common proteins between R- and G-DIMs, protein abundance was estimated using spectral counting, which is based on the cumulative sum of recorded peptide spectra that can match to a given protein [32]. Following the calculation of a normalized spectral abundance factor (NSAF) value for each protein across the four replicates, only six proteins displayed a significant (p < 0.05) differential accumulation between R- and G-DIMs (Figure 3C). Namely, a mitochondrial import receptor subunit TOM40 homolog, a fasciclin-like arabinogalactan protein and a hexokinase displayed a higher abundance in R-DIMs than in G-DIMs, whereas an elongation factor 1-alpha, a V-type proton ATPase subunit H and an asparagine synthetase over-accumulated in G-DIMs relative to R-DIMs. As a result, the 126 proteins shared between both fractions largely corresponded to a quantitatively conserved set of DIM-associated proteins irrespective of the extraction method, in which the top 10 major abundant proteins included transmembrane porins (aquaporins, OMP), respiratory chain related proteins (ATP synthases, flavoprotein), beta-glucosidase G1 and ubiquitin, as very often described in plant DIM fractions (Additional file 3: Table A2) [16,20]. On the opposite, the Venn diagram also showed that out of the 234 and 219 proteins identified in R- and G-fractions, 108 (46%) and 93 (42%) proteins were unique to R- and G-DIM, respectively (Figure 3D). This pointed out that 61% (201 proteins) of the 327 DIM-associated proteins in *M. truncatula* roots underwent a differential partition according to the purification procedure. Consequently, even though both approaches had equivalent protein extraction efficiencies, as inferred from the similar number of accessions identified in R-and G-fractions, they nonetheless displayed a differential selectivity toward microsomal proteins.

### DIM-enriched proteins differ between R- and G-fractions

To further assess the extent to which protein composition of R- and G-DIMs quantitatively differed from that of initial microsomes, an abundance ratio between NSAF values of DIM and Mic fractions was calculated for each protein. On this basis, accessions that reproducibly displayed at least a 2-fold higher abundance in R- and G-DIMs than in microsomes were considered as DIM-enriched proteins according to Borner’s *sensu*. Among them, 65 were unique to R-DIMs (fraction termed “R2xspecific”) and 46 were unique to G-DIMs (fraction termed “G2xspecific”), whereas 42 were shared between R- and G-DIMs (fraction termed “RG2xcore”) (Figure 3D). From these results, it was thus concluded that each extraction procedure generated a specific subset of DIM-enriched proteins from *Medicago* root microsomes, which accounted for 7.4 and 5.3% of the initial 874 identifications, for R- and G-protocols, respectively. The rest of study was thus essentially dedicated to the comparison of these two specific proteomes and the core subset, relative to the microsomal fraction.

When investigating the representation of previously published plant DIM-associated proteins within our proteomic data by using identification mapping tools and homology search against the protein listed in [6,7,13,15,33,34] and [20], 152 proteins already described in plant microdomains were identified within the total 327 R- and G- proteins, including 33 proteins usually referred to as canonical plant DIM markers in the literature such as remorin (Additional file 3: Table A2). Noticeably, 14 DIM markers were overall identified within DIM-enriched *Medicago* proteins, which encompassed fasciclin-like arabinogalactan proteins, hedgehog-interacting protein, receptor-like kinases, 14-3-3 like protein, phospholipase D, dynamins, and flotillin. However, their distribution remarkably differed between R2xspecific and G2xspecific subsets (Figure 3E), thereby comforting the view that R- and G-approaches displayed a differential selectivity toward certain classes of proteins.

Finally, to address whether known or putative non-membrane proteins might be enriched in R- and G- DIM fractions, we used, as a point of reference for *M. truncatula*, the rationale described by Daher and co-workers [30] that favors similarity search on the basis of which homologous proteins share the same location in many organisms, a strategy recognized more confident than the use of *in silico* algorithmic predictors for protein localization [13]. Consequently, DIM-enriched proteins obtained from R- and G-protocols were first compared with BLASTP to TAIR database accessions and were considered as membrane *M. truncatula* proteins when homologous sequences displaying at least 70% pair-wise identity and a cut-off expectation value of e^−40^ were experimentally demonstrated to have a membrane localization, including core integral or subunits of membrane complexes, on the basis of direct assays [30]. In the absence of TAIR homologues, LegumIP annotations that overall agreed up to 80% with Arabidopsis-inferred cellular components, even though largely less detailed, were used to address protein localization (Additional file 3: Table A2). In the absence of confident membrane homologues, DIM-enriched proteins were retained as non-membrane proteins unless predicted to display at least one of the following criteria: to form an alpha helical TM domain or a beta barrel embedded in the membrane lipid bilayer, to be anchored to the membrane owing to hydrophobic tails, and/or to be targeted to the secretory pathway, as previously described [11,35]. Using this design, 10 accessions mainly of cytosolic origin, out of the total 227 proteins previously recorded as DIM-enriched were identified as potential contaminants of membrane fractions (Additional file 3: Table A2). However, when considering their known or putative functional relevance in microdomain formation with special regard to role in mediating hydrophobic interactions and/or responses to microbial ingress/accommodation at the interface of plant-microbe interactions that largely depend on exocytocis, endocytosis, or local secretion of defense compounds [36], we made the deliberate choice not to discard them from R- and G-DIM fractions. Namely, patellin-5 binds to hydrophobic molecules such as phosphoinositides and promotes their transfer between different cellular sites. The PLAT/LH2 family domain of lipase/lipoxygenase is found in a variety of membrane or lipid associated proteins, and dynein transports various cellular cargo by walking along cytoskeletal microtubules. Ubiquitin, linkage of which to PM proteins is known to induce endocytosis and/or proteasome-dependent degradation [5], whereas caffeic acid 3-O-methyltransferase is involved in the reinforcement of the plant cell wall and in the responding to wounding or pathogen challenge by the increased formation of cell wall-bound ferulic acid polymers. Major latex proteins belong to cytokinin-specific binding proteins that also have role in pathogen defense responses. Sorting and assembly machinery component 50 **(**cell division protein FtsZ homolog) is part of a ring in the middle of the dividing cell that is required for constriction of cell membrane/cell envelope and localizes to very-long chain fatty acids-containing phospholipids that have an important role in stabilizing highly curved membrane domains [15,37]. Finally, glycoprotein-binding proteins (lectins) have been suggested to contribute to stimulus-dependent microdomain assemblies via cross-linking of PM-resident proteins [33,38].

Taken together, the above data confirmed that both the Rapid (R) and Gradient (G) protocols enabled the isolation of microdomain fractions directly from *M. truncatula* root microsomes, as inferred from sterol enrichment, presence of typical sphingolipid long-chain bases from plants, enrichment in membrane proteins including well-known plant DIM reporters, but also showed that the method used for DIM extraction, namely low-speed centrifugation *versus* floatation, qualitatively impacted the composition of the proteome enriched in DIM fractions relative to initial microsomes. Consequently, to get a deeper insight regarding the processes by which DIM-enriched proteins may preferentially partition to either R- or G-DIM fraction, the corresponding *M. truncatula* proteins were further characterized with regard to their subcellular localization, functional relevance and features known to drive membrane association.

### R- and G-DIM-enriched proteins differ in their cellular location

**Figure 4:**
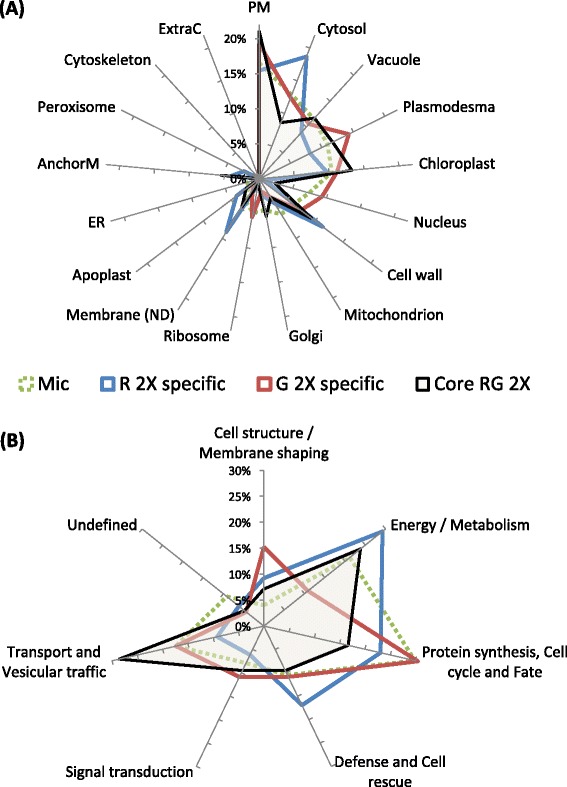
**Cellular (A) and functional (B) distribution of the 227 proteins recorded as enriched above 2-fold in DIM-fractions relative to microsomes of** ***M. truncatula*** **roots.** Subsets termed “R2xspecific” and “G2xspecific” refer to the proteins uniquely enriched in R-and G-DIMs, respectively, whereas “RG2xcore” designates the proteins enriched in both R- and G-DIMs, relative to microsomes. **(A)** Localization was inferred from TAIR and LegumeIP homologous proteins having experimentally checked cellular components. **(B)** Functional classification was performed using the FunCat scheme.

To analyze the subcellular localization of DIM-associated proteins of *M. truncatula* roots relative to the microsomal fraction, we used the above-described workflow that favors similarity searches over *in silico* predictions. Using these criteria, 23 different localizations were recorded for the 227 proteins enriched in the current DIM fractions, as detailed in Additional file 2: Table A1. In this respect, because chloroplast-located proteins in roots refer to those belonging to non-photosynthetic plastids, they were further termed non-green plastid proteins. To minimize misinterpretation, these 23 localizations were restricted to 17 after combining when possible each membrane fraction to its counterpart organelle, as for example plastids with plastidial membranes. Actually, although each cellular compartment was experimentally checked, reference to whole organelle localization can also include its membrane residents when not specifically addressed in the corresponding study. To take into account the multiple cell locations that a protein very often inhabits according to TAIR and LegumeIP annotations (Additional file 3: Table A2), a subcellular profile was thereby drawn for each of the R, G and microsomal fractions of interest by plotting the rate of occurrence of each cellular component within the corresponding proteomic data sets, as displayed in Figure 4A.

Keeping in mind that each frequency does not refer to an exclusive subcellular component and that frequencies may be biased toward the most studied Arabidopsis and legume organelles, it nonetheless appeared from Figure 4A that plasma membrane had the highest rate of occurrence within the RG2xcore proteome, a result that substantiates the view according to which the PM largely contributes to microdomain-enriched proteins [20]. However, although proteins ascribed to mitochondrion were largely depleted in this core fraction relative to initial microsomes, as previously observed by Zheng *et al.* [11], those located to other cellular components such as cell wall and non-green plastids happened to be enriched in *Medicago* root DIMs. Consequently, the subcellular profile obtained for this core fraction agreed with the idea that besides the plasma membrane, DIMs can be extracted from several other cellular compartments, as essentially demonstrated before for endomembrane systems when analyzing organelle-enriched fractions (Additional file 2: Table A1). In this respect, whereas the presence of plant cell wall-related proteins within DIM fractions has been widely reported in the literature [15,34], the retrieval of plastidial component in microdomains is far less documented. Nonetheless, *Arabidopsis* TOC75 protein, a component of the plastid outer membrane, was found in a fraction of detergent-insoluble membranes [20], supporting the idea that specific proteins might be included in microdomains of plastid membranes. In the current study, a beta-hydroxyacyl-(acyl-carrier-protein) dehydratase FabZ (Medtr2g008620), experimentally ascribed to the chloroplast envelope and the cell wall and reminiscent of the beta-hydroxyacyl-(acyl-carrier-protein) dehydratase precursor previously identified in *M. truncatula* DIMs [13], was enriched more than 50 fold in both R- and G-DIM fractions, relative to microsomes, Because this enzyme displayed no chloroplast transit peptide (cTP), but may be plastid-encoded according to HAMAP prediction (data not shown), it is likely that this protein that has role in lipid biosynthesis may serve specific function(s) at the plastid membrane. Likewise, phospholipase D alpha, a noticeable plant DIM marker that participates to the metabolism of phosphatidylcholines, which are important constituents of cell membranes, lipase/lipoxygenase, and patellin-5 (see above), also belonged to those lipid-related proteins co-enriched in R- and G-DIMs that can localize to non-green plastids (Additional file 3: Table A2). Regarding plastids, it is worth noting that these organelles are specialized, among other features, for the synthesis of fatty acid precursors that are either directly assembled within their own membranes, exported to the ER for extraplastidial lipid assembly, or reimported for the synthesis of plastidial lipids [39].

With special interest in those proteins specifically enriched in R- and G-DIMs relative to microsomes, the most remarkable differences recorded between the subcellular patterns of these two fractions included enrichment in proteins ascribed to cytosol/cell wall/undefined membrane components and a depletion of nuclear proteins in the R-specific subset, whereas plasmodesma- and nucleus-associated proteins were enriched in the G-specific fraction (Figure 4A). Among the 22 cytosolic proteins recorded as specifically enriched in R-DIMs, only 6 didn’t display any feature driving association to membranes and were exclusively assigned to cytosol according to experimental annotations, indicating that association of cytosolic proteins to R-DIM was not driven in the majority by non-membrane proteins. Likewise, all the 13 proteins located to the cell wall that were exclusively enriched in R-DIMs were predicted to have a membrane signature, as illustrated by germin-like protein, alpha-D-xylosidase, alpha-1,4-glucan-protein synthase, cysteine proteinase inhibitor 5, pectinesterase, beta-D-glucosidase, beta xylosidase, xylan 1,4-beta-xylosidase (Additional file 3: Table A2). It was also noticeable that R-procedure generated a DIM fraction largely depleted in nuclear proteins opposite to what observed for the gradient-based method, as previously depicted by Adam *et al.* [25]. Although mainly consisting of ribosomal proteins, most of the nucleus-ascribed proteins specifically enriched during G-DIM isolation displayed at least a membrane-related feature, which overall minimizes the likelihood that free ribosomes may have stricken to the lipid fraction during our extraction procedures [13]. Finally, plasmodesma-located proteins happened to be selectively enriched in G-DIMs, as inferred from the presence of 22 accessions ascribed to this compartment, although not exclusively, among which the DIM-marker flotillin belongs to (Additional file 3: Table A2). In plants, plasmodesmata correspond to membranous channels that allow intercellular communication. Embedded in the cell wall, they are defined by specialized domains of the endoplasmic reticulum and the plasma membrane, which may explain the large representation (Additional file 3: Table A2) of transporters and receptor-like kinase within the plasmodesma proteins enriched in G-DIMs, similar to what observed in the proteomic studies recently dedicated to plasmodesmata [40,41]. Due to the relative specialization of each organelle toward protein sorting and/or particular metabolic pathways, we thereby anticipated that the differential distribution of R- and G-DIM-enriched proteins over distinct cellular compartments might be of functional relevance.

### R- and G-DIM-enriched proteins differ in their functional relevance

**Figure 5:**
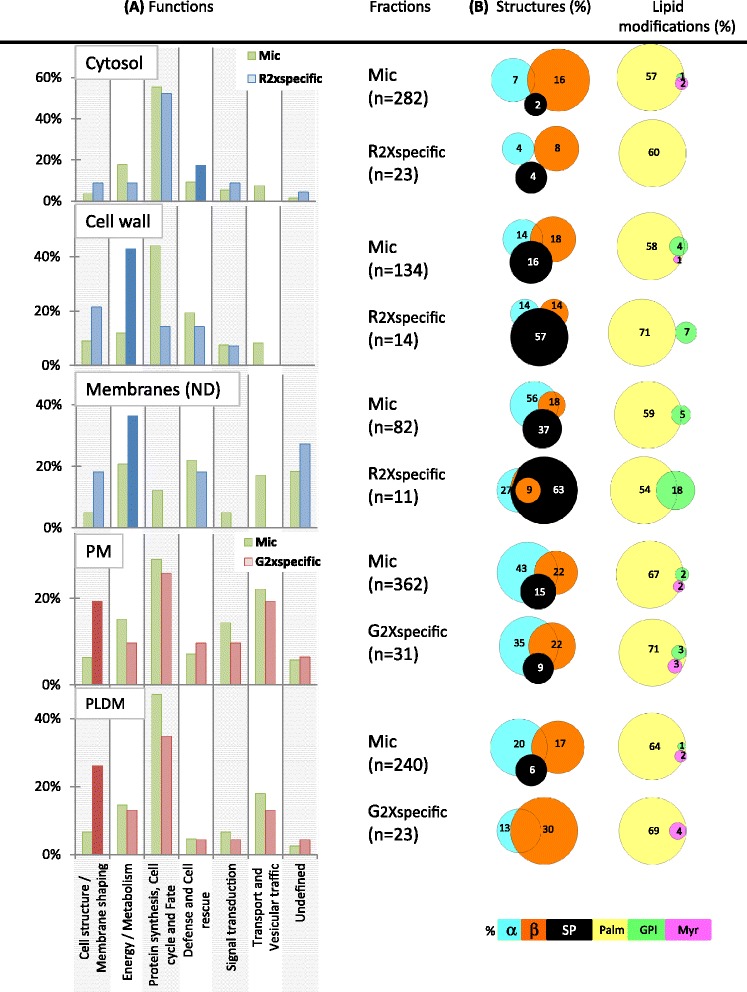
**Comparison of the distribution of (A) protein functional categories and (B) membrane-associative features between the cellular components enriched in R-DIMs (Cytosol, Cell wall, Membranes) and those enriched in G-DIMs (Plasma membrane, Plasmodesmata)**. Subsets termed “R2xspecific” and “G2xspecific” refer to the proteins uniquely enriched in R-and G-DIMs, respectively, relative to microsomes (Mic). The number (n) of proteins assigned to a given cellular component is indicated into brackets for Mic, “R2xspecific” and “G2xspecific” fractions **(A)** Functional distribution of the proteins ascribed to a cellular compartment enriched in R-DIMs (blue color) or G-DIMs (red color) relative to microsomes (yellow color), as inferred from the FunCat scheme. **(B)** Venn diagram distribution of membrane-associative features (%) within the proteins ascribed to a given cellular compartment, as predicted for protein sequence motifs (alpha-helices (blue), beta-strands (orange) and signal peptide (SP) (black) and lipid modifications (palmitoylation (yellow), GPI anchor (green), myristoylation (pink)).

To obtain an overview of the functionality of R- and G-DIM-enriched proteins relative to the microsomal proteome, the corresponding 874 identifications were classified according the FunCat annotation scheme [42] that assigned them to seven known biological processes. When regarding the fraction of the proteins co-enriched in R- and G DIMs, Figure 4B showed a noticeable increase in the “transport and vesicular traffic” category, which is consistent with anterior repertoires showing that DIM-associated proteins are largely described in the context of membrane transport [43,44]. Signal transduction related-proteins were also enriched in this core proteome, but to a lesser extent than expected from what usually observed in animal systems. This feature previously reported for DIMs extracted from the PMs of oat and rye, but also within sterol-dependent proteins of Arabidopsis, thereby comforts the opinion that not all signaling proteins are necessarily enriched in microdomains [15,34]. Remarkably, Figure 4B also indicated that different functional categories were preferentially represented depending on the protocol used. Namely, proteins playing roles in “proteins synthesis/fate” were depleted in R-DIMs, relative to microsomes. Not observed for G-DIMs, this later pattern was consistent with the depletion of nuclear ribosomal proteins noticed in the current study and by Adam *et al.* [25]. Additionally, opposite to the functional partitioning obtained with the gradient method, proteins ascribed to “defense/cell rescue” and “energy/metabolism” processes were specifically enriched in R-DIMs. Finally, the category related to “cell structure/membrane shaping” displayed a larger increase within those proteins enriched in G-DIMs than after R-mediated sedimentation. To check whether the distribution of these functional categories could mirror the differential partitioning of cellular components in each DIM fraction, we then plotted the rate of occurrence of the Funcat-ascribed functions within the proteins specifically-enriched in each organelle, relative to microsomes. Figure 5 showed that cytosolic and cell wall/membrane proteins typical of R-DIMS were essentially involved in defense/cell rescue and energy/metabolism, respectively. By contrast, the PM and plasmodesma-located proteins that were preferentially recruited in G-DIMs mostly played role in cell structure/membrane shaping processes. In a previous work [13], proteins sustaining cellular trafficking and cell wall functioning were also found well-represented in DIM fractions prepared from *M. truncatula* root plasma membrane. The latter two functional categories, together with that corresponding to biotic/abiotic stress responses, were highlighted in DIM recovered from tobacco PM [6].

Taken together, the above-data showed that despite the existence of a conserved core of proteins, R- and G-protocols each resulted in the enrichment of a particular DIM-proteome displaying specific cellular localizations and biological functions. Differences in protein and/or lipid associated with DIM fractions have been reported earlier, but essentially as dependent upon extraction parameters such as temperature, concentration and type of detergent [45,46]. However, in the current study, DIM extraction procedures (temperature, duration, and detergent) were identical for both R and G protocols, which consequently only differed at DIM isolation process, namely sedimentation *versus* floatation on sucrose gradient. In this context, it was reasonable to assume that the selectivity displayed by each method could have arisen from some particular membrane compatible characteristics displayed by these differentially-enriched proteins.

### Membrane-associative features as related to R/G protein partitioning

Among the features favoring protein embedment in the hydrophobic lipid bilayer are membrane spanning protein domains that include typically alpha-helices or beta-sheets with hydrophobic surfaces serving as the interface to the hydrocarbon core of the lipid bilayer. In addition to, signal peptides of nascent proteins can also mediate protein translocation across or integration to membranes along the secretory pathway [47]. Protein association to membranes can also be driven by lipidic anchors, which can either be permanent co-translational additions or posttranslational modifications. These lipid modifications include glycophosphatidylinositol (GPI) anchors, N-terminal myristic acid tails (myristoylation), and cysteine acylation (palmitoylation) [35]. Consequently, we further monitored and compared the putative presence of *trans*-membrane (TM) spanning domains, signal peptide (SP) sequences, and lipid modifications and within R- and G-enriched proteins, relative to microsomes, as inferred from the corresponding online predictor tools (see Methods).

**Figure 6:**
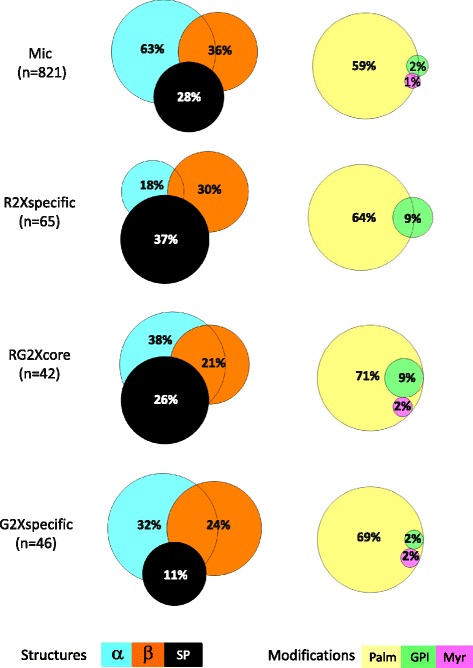
**Comparison of Venn diagram distribution of predicted membrane-associative features (%) between the “R2xspecific”, “G2xspecific”, “RG2xcore”, and Mic protein subsets, as related to protein sequence motifs (alpha-helices (blue), beta-barrels (orange) and signal peptide (SP) (black) and lipid modifications (palmitoylation (yellow), GPI anchor (green), myristoylation (pink)).** The number (n) of proteins contained within each diagram is indicated into brackets. Subsets termed “R2xspecific” and “G2xspecific” refer to the proteins uniquely enriched in R-and G-DIMs, respectively, relative to microsomes (Mic), whereas “RG2xcore” designates the proteins enriched in both R- and G-DIMs, relative to microsomes.

With regard to the distribution of lipid modifications within each of the cellular components specifically enriched in R (cytosol, CW, undetermined membranes) and G-DIMs (PM, plasmodesmata), Figure 5 indicated that the proportion of putative palmitoylated proteins was not affected by either of the method used. This result also holds true when considering the total subsets of the proteins enriched in R- and G- fractions (Figure 6). Overall, S-palmitoylation largely prevailed as the most abundant putative lipid modification associating proteins to membrane compartments in *M. truncatula*, as predicted for 487 (59%) candidates within the total microsomal fraction. This result is consistent with recent proteomic data derived from root callus culture of Arabidopsis, in which the number of proposed S-acylated proteins has increased from 30 to over 500 [48]. By contrast, GPI anchors that seemed more frequently predicted for R-enriched proteins than for G, relative to microsomes, only encompassed a few number of proteins (Figures 5 and 6). Likewise, the presence of putative N-terminal myristic acid tails was anecdotic within the proteins enriched in R- and G-DIMs. It appeared from Figure 5 that the most noticeable difference between the two proteomes corresponded to an increase in the proportion of predicted SP-containing proteins after sedimentation (R) compared to its decrease after floatation (G). This behavior, which was also observed at a larger scale when looking at the total subsets of the proteins enriched in R- and G- fractions (Figure 6), indicated that part of the late secretory pathway may likely contribute to the specificity of the R-DIM proteome. The classical eukaryotic pathway for secretion includes translocation of nascent proteins into the ER lumen and then through the secretory pathway, which comprises the Golgi apparatus (GA) and *trans*-Golgi network, protein packaging into vesicles that migrate to, and fuse with the PM, releasing the protein cargo into the cell wall, or are targeted to the vacuole or other post-Golgi compartments [49]. The default pathway for proteins with signal peptides and with no additional targeting information is to proceed through the ER, Golgi, and PM where they are secreted into the cell wall. It is noteworthy that for targeting to the PM, proteins do not necessary require a signal peptide in so far as additional sequences, including membrane spanning regions, also allow them to flow to the PM [50]. Consequently, the preferential partitioning of SP-predicted proteins within the cell wall and endomembrane components specifically enriched in the R-fraction suggested that DIMs containing proteins targeted to secretion might not have a buoyancy similar to those uniquely retrieved by floatation (e.g., mainly PM and plasmodesmata DIMs). Consistent with this possibility, when investigating the distribution of microdomains in leek plant cells, Laloi *et al.* [16] showed that replacement of usual ∆^5^-sterols induced a preferential formation of DIMs in the GA compared to the PM, indicating that sterols didn’t contribute equally to DIM formation along the secretory pathway. There were additional evidences for a preferential accumulation of sterols in the PM, with a progressive increase through the secretory pathway both in *Zea mays* and *Allium porrum* [51,52]. Remarkably, inhibition of sterol biosynthesis also led to a decrease in DIM protein markers within the PM without affecting vesicular transport to the cell surface, suggesting a likely requirement of particular sterols for protein targeting to PM microdomains [16,53]. In *M. truncatula* roots, spinasterol was reported as the dominant compound in DIMs prepared from the PM [13], and, as described above, we also demonstrated that this sterol was enriched to a larger extent in G-than R-fractions. Inferred from these results and from the preferential retrieval of PM-related proteins in G-DIMs relative to R-fractions in which protein secretion dominates, we thus assume that spinasterol enrichment participates to the buoyancy of PM and plasmodesma DIMs, opposite to the DIMs located along the late secretory pathway in which sterols are suspected to be less concentrated [16].

**Figure 7:**
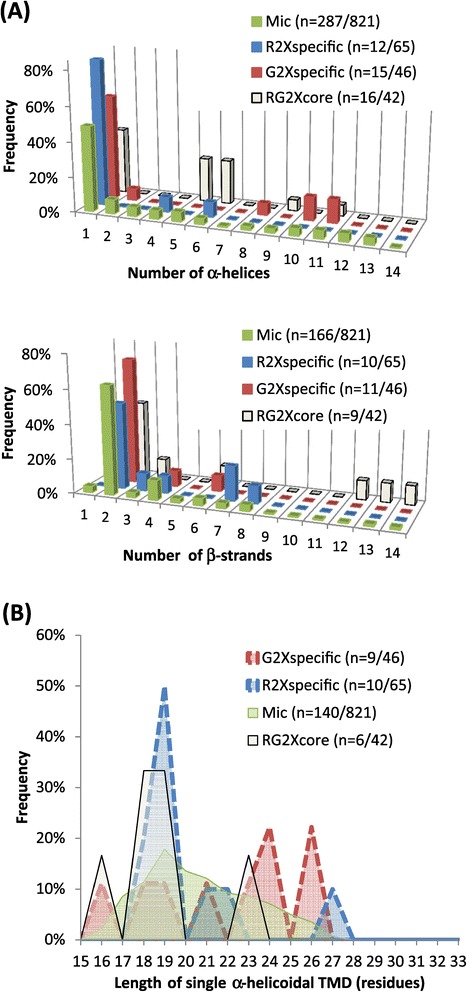
**Comparison of the characteristics of the predicted** ***trans*** **-membrane domains between the “R2xspecific”, “G2xspecific”, “RG2xcore”, and Mic protein subsets. (A)** Distribution (%) of the number of alpha-helices (top panel) and beta-strands (bottom panel). **(B)** Distribution (%) of the length of single alpha-helicoidal spanning domain. Subsets termed “R2xspecific” and “G2xspecific” refer to the proteins uniquely enriched in R-and G-DIMs, respectively, relative to microsomes (Mic), whereas “RG2xcore” designates the proteins enriched in both R- and G-DIMs, relative to microsomes. The number (n) of involved proteins is indicated into brackets for each fraction.

In a similar line of reasoning, it has been shown that sterol enrichment within the PM can increase its hydrophobic thickness relative to ER and Golgi endomembranes, thereby mediating the selective targeting of proteins that have corresponding TM hydrophobic length [54,55]. This process, referred to as the bilayer-mediated mechanism [16], mirrors the view that due to their high concentrations of sterols and sphingolipids with long, saturated hydrocarbon chains, microdomains may have thicker bilayers than the surrounding lipid matrix containing unsaturated phospholipids [56]. Consequently, proteins with relatively long TM hydrophobic regions would be expected to localize in the thick bilayers, whereas shorter TM proteins should localize in the thinner non-raft regions [57,58]. In the current study, analysis of TM domain distribution, as displayed in Figure 6, indicated a substantial depletion of predicted alpha-helices in the proteins enriched within microdomain fractions relative to microsomes, which dropped from 63 to 18 and 32% in R- and G-DIMs, respectively. This pattern, which was also observed when considering DIM-enriched cellular compartments with the exception of cell wall-related proteins (Figure 5), is consistent with the view that the presence of TM domains *per se* is not a prerequisite to drive protein association to microdomains [35]. When refining the partitioning of TM domains with special attention to their number and length that may contribute to both organelle localization and microdomain affinity, it turned out that relative to the microsomal fraction, proteins predicted to be anchored by a single membrane-spanning helix were largely enriched in both DIM fractions, and to a larger extent in R-(83%) than in G-(60%) DIMs (Figure 7A). One rationale for the recruitment of single*-*span TM proteins within DIMs, as reported earlier in the PM microdomains of tobacco and barrel medic [6,13], may be that oligomerization of monomeric TM proteins would increase their affinity for microdomains, which otherwise would only display a short residency time within rafts [59]. When examining the length of these single*-*span TM domains, Figure 7B showed that although helices comprising 19 amino acids tended to be more frequently enriched in R-DIMs relative to G-DIMs in which stretches of 24 and 26 amino acids dominated, these differences may not be of significant relevance as they only encompassed a very few number of proteins. *In silico* analysis of the number of TM domains also indicated that proteins predicted to contain eight to twelve alpha-helices were enriched in G-DIMs but not in R-DIMs, relative to microsomes (Figure 7A top panel). This feature that was previously highlighted in the PM microdomains of tobacco [6], therefore agrees with the preferential recruitment of plasmalemma-located proteins within G-DIMs (Figure 4A). Finally, we remarked that dynamin-2B, flotillin, stomatin and leucine-rich repeat (LRR) proteins belonged to those membrane-localized proteins predicted to contain 2 beta-strands, which were enriched in G- but not in R-DIMs relative to microsomes (Figure 7A bottom panel). In this regard, dynamin-2B, flotillin and stomatin can display a hairpin-like topology, susceptible to influence membrane curvature and scaffolding processes in microdomains [60]. Likewise, LRR have curved horseshoe structures that are known to drive protein-protein-interactions [61] and can also act as a raft nanodomain targeting signal [62]. Overall, *in silico* predictions showed that R- and G-DIM-enriched proteins differ in the distribution of the three protein motifs currently investigated that can drive association to membrane compartments, namely alpha-helices, beta-strands, and signal peptides. The preferential partitioning of predicted SP-containing proteins within the cell wall and endomembrane components specifically enriched in R-DIMs, suggested that part of the late secretory pathway may contribute to the specificity of the R-DIM proteome.

## Conclusions

In the current study, DIMs were prepared for the first time directly from *M. truncatula* root microsomes that consist of a complex membrane mix relative to the PM conventionally used as starting material for microdomain isolation. We clearly established that both long-lasting sucrose gradient centrifugation (G protocol) and rapid microfuge sedimentation at low speed (R protocol) enable the recovery of membrane fractions that meet the criteria of DIMs, as inferred from sterol enrichment, presence of typical sphingolipid long-chain bases from plants, and enrichment in membrane proteins including canonical DIM markers. Proteomic analysis of the corresponding fractions also show that, despite the existence of a conserved core of proteins, R- and G-protocols result in the enrichment of a particular DIM-proteome displaying specific cellular localizations and biological functions. Collectively, even though microsomes were used as initial material, we show that the composition of the G-DIM fraction still mostly mirrored that of PM microdomains conventionally retrieved by floatation. In parallel, the possibility to isolate by rapid differential centrifugation a DIM fraction that seems to target the late secretory pathway opens new avenues to study plant microdomains. Finally, with regard to our initial questioning addressing the intracellular distribution of plant DIMs, current results obtained in *M. truncatula* roots clearly support the existence of microdomains not only in PM and the late secretory pathway, but also in additional membrane organelles, including non-green plastids.

## Methods

### Plant material

*Medicago truncatula* cv. Jemalong 5 seeds were surface-sterilized and germinated at 27°C in the dark onto 0.7% sterile agar [21]. Two-day old seedlings were then transferred on soil and grown into 400 ml plastic pots containing a mix of sterile soil of Epoisses (neutral clay loam from Domaine d’Epoisses, INRA Dijon France) and sand (1:2, v/v) supplemented twice a week with a nitrogen-enriched nutrient solution (Long Ashton [63]) under controlled conditions (16 h photoperiod, 220 μE.m^−2^.s^−1^ light irradiance). After 4 weeks, roots were collected, gently rinsed with deionized water to get rid of soil, deep frozen in liquid nitrogen and stored at −80°C until further use.

### Microsomal protein purification

All steps of microsome preparation were carried out at 4°C according to [64]. Microsomes of *M. truncatula* roots were obtained by differential centrifugation as previously described for tobacco cells [7]. Briefly, frozen roots (about 100 g fresh weight) were homogenized using a Waring Blender in grinding buffer (50 mM Tris-MES, pH 8.0, 500 mM sucrose, 20 mM EDTA, 10 mM DTT and 1 mM PMSF). The homogenate was successively centrifuged at 12.000×*g* and 16.000×*g* for 20 min. After centrifugation, supernatants were collected, filtered through two successive meshes (63 and 38 μm), and centrifuged at 100.000×*g* for 1 h. Microsomal pellets were resuspended in buffers according to the DIM fraction isolation procedure used (see below), homogenized with a glass pestle. Protein contents were quantified using the RCDC (bicinchoninic acid) Protein Assay Kit (BioRad) to avoid TX-100 interference, using BSA as standard.

### Detergent-insoluble-membrane fraction isolation

For both the Rapid (R) and the Gradient (G) protocol (Figure 1), three independent extractions of DIMs were performed, each from a 6 mg protein equivalent of microsomes. Anotations R/G x:y were used to refer to the R or G protocol, the x detergent/protein ratio (w/w), and the y (%, v/v) final detergent concentration, respectively.

The rapid protocol was performed as described in [25]. The microsomal fraction was first resuspended in 10 mM Tris-MES buffer pH 7.3, containing 250 mM sucrose, 1 mM EDTA, 10 mM DTT, 1 mM PMSF, 10 μg/ml aprotinin and 10 μg/ml leupeptin. To increase solubilisation, resuspended microsomal proteins were aliquoted into 1 mg fractions that were further diluted (270 μl final volume) with 25 mM Tris-MES pH 6.5, containing 150 mM NaCl. Then, 30 μl of 10% (v/v) Triton X-100 was added to reach a final detergent-to-protein ratio of 3:1 (w/w) and a 1% final detergent concentration (v/v). Incubation was performed on ice under gentle shaking for 30 min. Samples were centrifuged at 16.000x*g* for 20 min. The resulting Triton X-100-insoluble fraction (DIM) was homogenized (using a micropestle) in 200 μl of beta-octylglucoside-containing buffer (60 mM beta-octylglucoside, 10 mM Tris HCl pH7.5, 150 mM NaCl) and incubated on ice under gentle shaking for 30 min. A last centrifugation step (16.000×*g* for 20 min) was performed to separate soluble DIM fraction (R-DIM) from both Triton X-100 and beta-octylglucoside-insoluble pellet. Aliquoted DIM fractions were pooled and protein amount was measured using the RCDC Protein Assay Kit (BioRad).

For the gradient protocol, the microsomal pellet was resuspended in TNE buffer (25 mM TrisHCl pH 7.5, 150 mM NaCl, 5 mM EDTA) according to Borner *et al.* [20]. The microsomal fraction (6 mg protein equivalent) was treated with 1% (w/w) Triton X-100 for 30 min at 4°C under gentle shaking. After solubilization, membranes were brought to a 1.8 M sucrose final concentration (using a 2.4 M sucrose/TNE buffer), overlaid with 2 ml of 1.6 M, 1.4 M, 1.2 M and 0.15 M sucrose in TNE buffer, and then spun for 16 h at 200.000×*g* at 4°C. DIMs were collected at the 1.2-1.4 M sucrose interface, washed with an excess of TNE buffer and centrifuged at 100.000×*g* for 1 hour to remove residual sucrose. The DIM pellet (G-DIMs) was homogenized in 1 ml of beta-octylglucoside containing buffer (60 mM beta-octylglucoside, 10 mM Tris HCl pH7.5, 150 mM NaCl). Protein concentration was determined with the RCDC Bio-Rad protein assay.

### Free sterol extraction and analysis

Total lipids were extracted from microsomal and DIM fractions (R and G; 100 μg equivalent proteins per sample, previously diluted in 0.37 M KCl to reach a final volume of 500 μl) according to Folch *et al.* [65] with chloroform/methanol (2:1, v/v). The extraction of lipids was carried out in 25 ml glass tubes with Teflon lined screw caps. Sterol internal standard (10 μg epichoprostanol) was added to Mic, R and G samples but not into one Mic sample (Mic-Std). All solutions were then mixed with MetOH/chloroform 2:1 (v/v), shaken and left overnight at 4°C. The next day, chloroform was added to reach a final ratio MetOH/chloroform 2:4 (v/v) and the mixture was centrifuged at 320×*g* for 8 min. The sterol-containing lower phase was collected, dried under nitrogen flux, washed once with EtOH and dried again. Lipids were saponified using ethanolic KOH (1 ml EtOH, 100 μl KOH (11 N)). Upon warming at 80°C for 1 h, 2 ml H_2_0 and 2 ml hexane were added and the lipids were recovered by centrifugation (320×*g*, 8 min). The upper phase was evaporated to dryness under nitrogen flux. Then, the residue was derivatized by adding 100 μl of the silylating agents (BSTFA/TMCS mixture (5:1 v/v)) and warmed at 80°C for 1 hour. Samples were finally analyzed for their sterol content by gas chromatography after addition of 400 μl hexane.

Gas chromatography analyses were carried out on Agilent 7890 GC instrument equipped with a with a flame ionization detector, on a Varian Factor Four VF-5 ms capillary column (15 m, 0.32 mm i.d. × 0.25 μm film thickness). Sample manual injection (1 μl) was performed in splitless mode (split vent at 30 seconds; injector temperature 240°C). Helium was the carrier gas at 1.5 ml/min in constant flow mode. Temperature program was programmed from 120°C to 240°C at 9°C/min. Data were processed using the Agilent EZ Chrom Elite software providing retention time and area for each compound of interest.

### Quantification of sphingolipid long-chain bases

LCB content of DIM and microsomal fractions were determined as previously described Cacas *et al.* [26] from three individual experiments. Briefly, LCB were released from fractions by direct overnight incubation at 110°C in 1 ml dioxane and 1 ml 10% (w/v) Ba(OH)_2_ solution prepared in water. Before incubation, standard LCB (d14:1, d17:1 and d20:0) used for quantification were directly added to the dioaxane/barium mixture (10 μg for each standard LCB/sample). Upon cooling and addition of 6 ml distilled water, LCB were extracted twice with 4 ml diethylether. Pooled diethylether phases were dried under nitrogen flux. Dry residues were dissolved in 1 ml methanol containing 100 μl of a freshly prepared 0.2 M metaperiodate (NaIO_4_) solution. Oxidation of extracted LCB into aldehydes was then carried out in the dark for 1 hour at room temperature and under mild shaking, as described by Kojima *et al.* [66]. LCB-derived long-chain aldehydes were extracted into 1 ml hexane following addition of 1 ml water. To concentrate samples, the aldehyde-containing hexane phase was dried under nitrogen flux, and aldehydes were finally resuspended in 100 μl hexane to be injected into GC-MS.

For the separation of LCB-derived fatty aldehydes, a 30 m × 250 μm HP-5MS capillary column (5% phenyl-methyl-siloxane, 0.25 μm film thickness, Agilent) was used with helium carrier gas at 2 ml/min; injection was in splitless mode; injector and MS detector temperatures were set to 250°C (Agilent 6850 coupled to a mass analyzer Agilent 6975); the oven temperature was held at 50°C for 1 min, then programmed with a 25°C/min ramp to 150°C (2 min hold), a 10°C/min ramp to 210°C, and 75°C/min ramp to 320°C (5 min hold). Upon separation by GC and detection by MS, fatty aldehydes were identified based on their retention time and fragmentation [26]. The ion current of each molecular species of interest was determined and further used for calculating the amount of molecules by comparison with the appropriate internal standards. These results were expressed in micrograms. Taking into account the molecular weight of individual LCB-derived aldehydes, the quantity in moles for each molecular species was calculated and expressed as mole%.

### One-dimensional SDS-PAGE and nano-LC-MS/MS analysis

Microsomal and DIMs samples (20 μg protein equivalent) were mixed at a ratio of 1 to 1 with Laemmli buffer [67] without any heating denaturation step. Samples were separated onto small 12% polyacrylamide gels with 4.5% stacking gel and proteins were stained with colloidal blue (G250). Proteins were separated along a short (1 cm)-migration. Individual gel lanes were sliced in 7 pieces for in-gel digestion and LC-MS/MS analysis. Each section was washed in water and completely destained using 100 mM NH_4_CO_3_ in 50% acetonitrile (ACN). A reduction step was performed by addition of 100 μl of 50 mM NH_4_CO_3_, pH 8.9, and 10 μl of 10 μM TCEP (Tris(2-carboxyethyl) phosphine HCl) at 37°C for 30 min. The proteins were alkylated by adding 100 μl of 50 mM iodoacetamide and allowed to react in the dark at 20°C for 40 min. Gel sections were first washed in water, then ACN, and finally dried for 30 min. *In-gel* digestions were performed with trypsin in the Progest system (Genomic Solution, East Lyme, CT, USA) according to a standard protocol. Gel pieces were washed twice by successive baths of 10% (v/v) acetic acid, 40% (v/v) ethanol and ACN. They were then washed twice with successive baths of 25 mM NH_4_CO_3_ and ACN. Digestion was subsequently performed for 6 h at 37°C with 125 ng of modified trypsin (Promega) dissolved in 20% (v/v) methanol and 20 mM NH_4_CO_3_. Peptides were extracted successively with 2% (v/v) TFA and 50% (v/v) ACN and then with pure ACN. Peptide extracts were dried and suspended in 20 μl of 0.05% (v/v) TFA, 0.05% (v/v) HCOOH, and 2% (v/v) ACN.

Mass spectrometry analysis was carried out on 2 independent replicates for each DIM fraction (R and G). Peptide separation was performed using an Eksigent 2D-ultra-nanoLC (Eksigent Technologies, Livermore, CA, USA) equipped with a C18 column (5 μm, 15 cm × 75 μm, PepMap, LC packing). The mobile phase consisted of a gradient of solvents A 0.1% HCOOH (v/v) in water and B 99.9% ACN (v/v), 0.1% HCOOH (v/v) in water. Peptides were separated at a flow rate of 0.3 μl/min using a linear gradient of solvent B from 5 to 30% in 60 min, followed by an increase to 95% in 10 min. Eluted peptides were online analysed with a LTQ XL ion trap (Thermo Electron) using a nanoelectrospray interface. Ionization (1.5 kV ionization potential) was performed with a liquid junction and a non-coated capillary probe (10 μm i.d.; New Objective). Peptide ions were analyzed using Xcalibur 2.0.7, with the following data-dependent acquisition steps [68] full MS scan (mass to charge ratio (*m/z*) 300–2000, centroid mode), (2) MS/MS (qz = 0.25, activation time = 30 ms, and collision energy = 35%, centroid mode). Step 2 was repeated for the three major ions detected in step 1. Dynamic exclusion was set to 45 s.

### Protein identification and quantification

Searches were performed using the Mascot search engine (http://www.matrixscience.com) on the *Medicago truncatula* pseudomolecule database (http://www.jcvi.org/cgi-bin/medicago/annotation.cgi) version 3.5v3 (47529 entries). Trypsin digest was set to enzymatic cleavage, carboxyamidomethylation of, and oxidation of methionines were defined as fixed and variable modifications, respectively. Precursor mass precision was set to 2.0 Da with a fragment mass tolerance of 0.5 Da. Sequences corresponding to keratins or trypsin were removed by querying a homemade contaminant database as a first step of filtration. Identified proteins were validated according to the presence of at least two peptides with an E value smaller than 0.05. To take redundancy into account, proteins were grouped according to the Legoo server (http://www.legoo.org/).

Quantification of proteomic data was achieved by normalized spectral abundance factor (NSAF) analysis [32]. As NSAF represent percentages, all data were arsin square root-transformed to obtain a distribution of values that could be checked for normality [69] by using the Kolmogorov-Smirnov test at a 95% confidence interval. The protocol effect on protein abundance was analyzed by the Student’s *t*-test using the XLSTAT software package.

### *In silico* predictions

Alpha-helical TM spans and signal peptides were predicted according to the Phobius algorithm (http://phobius.sbc.su.se), whereas the online tool (http://biophysics.biol.uoa.gr/PRED-TMBB/input.jsp) was employed to discriminate *trans*-membrane beta-strand protein domains. N-myristoylation, S-palmitoylation and GPI anchor predictions were inferred from (http://mendel.imp.ac.at/myristate/SUPLpredictor.htm), (http://csspalm.biocuckoo.org/online.php), and (http://gpi.unibe.ch/), respectively. Close homologues of the identified proteins in *Medicago* were searched against The Arabidopsis Information Resource [70] (http://www.arabidopsis.org/) database with at least 70% pair-wise identity and a cut-off expectation value of e^40^. When necessary, ChloroP (http://www.cbs.dtu.dk/services/ChloroP/) was used to predict cTP, and homologies were searched within the HAMAP database (http://www.pdg.cnb.uam.es/cursos/Leon_2003/pages/visualizacion/programas_manuales/spdbv_userguide/us.expasy.org/sprot/hamap/families.html) that takes stock of plastid genome-encoded proteins.

### Availability of supporting data

The data sets supporting the results of this article are included within the article and its additional files.

## Additional files

Additional file 1: Figure A1. Influence of Triton X-100 concentration on lipid pattern. Additional file 2: Table A1. List of the 874 nonredundant proteins identified in all fractions. Additional file 3: Table A2. List of the 327 proteins identified in either Rapid (R) or Gradient (G) DIM fractions.
