# Supplementary material for: Direct purification of detergent-insoluble membranes from Medicago truncatula root microsomes: comparison between floatation and sedimentation
Source: BMC Plant Biol. 2014 Sep 30;14:255. doi: 10.1186/s12870-014-0255-x (PMC4193990; doi:10.1186/s12870-014-0255-x)
Supplement: Additional file 1: Figure A1. — Influence of Triton X-100 concentration on lipid pattern. [file 12870_2014_255_MOESM1_ESM.pptx]

## Slide 1
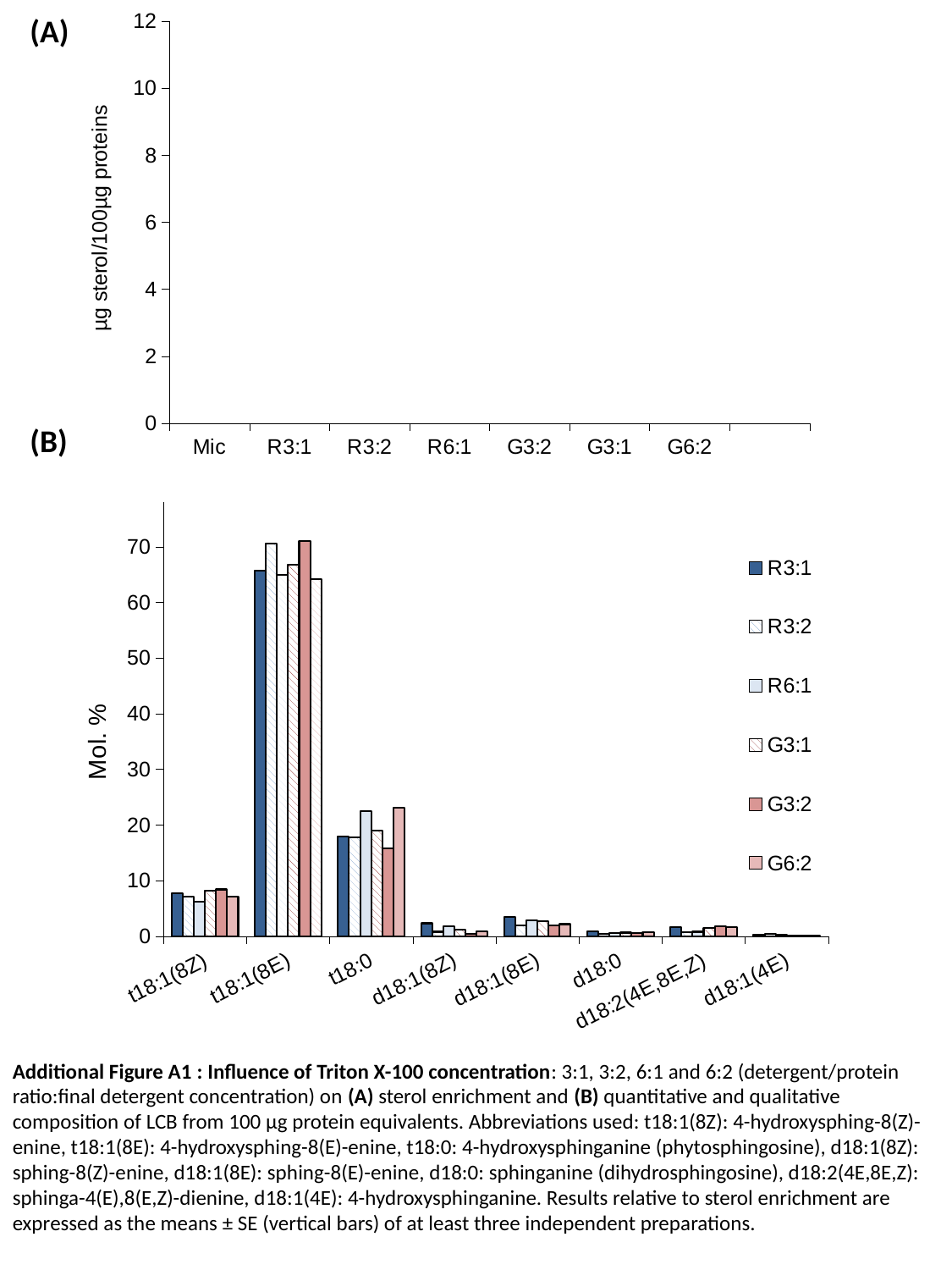

(A)
### Chart
| Category | Q sterols |
|---|---|
| Mic | 2.6646934158976694 |
| R3:1 | 7.591479463224606 |
| R3:2 | 6.484313010356807 |
| R6:1 | 6.772323850857617 |
| G3:2 | 10.722883420679898 |
| G3:1 | 10.695007530230837 |
| G6:2 | 10.029801899963765 |(B)
### Chart
| Category | | | | | | |
|---|---|---|---|---|---|---|
| t18:1(8Z) | 7.780092307642706 | 7.193196784512399 | 6.193436232356989 | 8.137868106090279 | 8.414265305776716 | 7.125215734150813 |
| t18:1(8E) | 65.78036056860263 | 70.62993306551827 | 65.01299670876168 | 66.74168747723253 | 71.0667270326967 | 64.26248113152143 |
| t18:0 | 17.89360673488255 | 17.813325285259197 | 22.52391186583542 | 18.930871356992466 | 15.789993213753034 | 23.10039709611647 |
| d18:1(8Z) | 2.328943650438873 | 0.8142399868660606 | 1.8564632099452474 | 1.2192332597410214 | 0.4055326994222486 | 0.8204229511004382 |
| d18:1(8E) | 3.4565062672999276 | 1.8942230483862335 | 2.8222960764672598 | 2.7665834381385994 | 1.897075290329304 | 2.178132994498677 |
| d18:0 | 0.9131673757056414 | 0.45550367532253666 | 0.5332243100441991 | 0.6596053565998897 | 0.5283384714385831 | 0.7685841452774775 |
| d18:2(4E,8E,Z) | 1.59648780823225 | 0.7799874582467032 | 0.8135301278633768 | 1.4752808703367788 | 1.839429309024479 | 1.6605605256604774 |
| d18:1(4E) | 0.2508352871954099 | 0.41959069588859615 | 0.2441414687258402 | 0.06887013486841648 | 0.05863867755891628 | 0.08420542167421355 |Additional Figure A1 : Influence of Triton X-100 concentration: 3:1, 3:2, 6:1 and 6:2 (detergent/protein ratio:final detergent concentration) on (A) sterol enrichment and (B) quantitative and qualitative composition of LCB from 100 µg protein equivalents. Abbreviations used: t18:1(8Z): 4-hydroxysphing-8(Z)-enine, t18:1(8E): 4-hydroxysphing-8(E)-enine, t18:0: 4-hydroxysphinganine (phytosphingosine), d18:1(8Z): sphing-8(Z)-enine, d18:1(8E): sphing-8(E)-enine, d18:0: sphinganine (dihydrosphingosine), d18:2(4E,8E,Z): sphinga-4(E),8(E,Z)-dienine, d18:1(4E): 4-hydroxysphinganine. Results relative to sterol enrichment are expressed as the means ± SE (vertical bars) of at least three independent preparations.
